# Mr. Adams on the Origin of One of His Instruments and Operations for the Cure of Cataract

**Published:** 1813-02

**Authors:** William Adams

**Affiliations:** Member of the Royal College of Surgeons in London, and the late Surgeon to the West-of-England Infirmary for curing Diseases of the Eye, instituted at Exeter. 28, Albemarle-street


THE
### Medical and Physical Journal.

2 OF VOL. XXIX.] FEBRUARY, 1813. [NO. 168.

*For the Medical and Physical Journal.*

### MR. ADAMS *on the* ORIGIN *of one of his* INSTRUMENTS *and* OPERATIONS *for the* CURE *of* CATARACT.

I HAVE always considered it to be the duty of a professional man, who, from the opportunities afforded by an extensive public and private practice, has been enabled to make improvements in medicine or surgery, to communicate the result of his experience to his professional brethren, for the general benefit of mankind. So few, comparatively, enjoy those opportunities, that it becomes the more incumbent on them to make no concealment of any useful knowledge acquired by such advantages. Those, therefore, who have deviated from this honorable line of conduct, have usually been assailed with well-merited reproaches. Every prudent man will, however, be careful, for the sake of his own reputation, to avoid the disclosure of a new practice, until, by repeated and varied trials, he has ascertained its comparative merits with that which is generally adopted; and he is justly entitled to be tenacious of a premature communication of his opinions, least the credit of his own invention should be seized upon by another, or its value, by a partial anticipation, be depreciated in the public estimation before it is thoroughly understood. Circumstances may sometimes give to this sanctionable caution an appearance of unnecessary concealment, and expose the most deserving individual to severe and unmerited censure. I allude more particularly to my late friend and preceptor Mr. Saunders.

In my practical observations on Ectropium, artificial pupil, and cataract, I have had occasion to notice the accusations of illiberal concealment, which since his death have been brought against him, and have there expressed my gratitude for his disinterested and liberal conduct to me, which conduct I brought forward as a direct refutation to those accusations, and now feel happy in the opportunity of repeating. His first attentions to me originated in consequence of our having served an apprenticeship to the same general practitioner, the late Mr. Hill, of Barnstaple, whose successful practice on diseases of the eye gave to us both a predilection for that branch of our profession. I had no claims on the friendship of Mr. Saunders, yet he not only gratuitously instructed me in the general principles of his practice, and allowed me for nearly eighteen months to assist him in the whole of his operations both on his public and private patients, but so entirely free was his mind from the least tincture of professional illiberality, that he afterwards assisted me by the most friendly services in the establishment of an institution precisely similar to his own, allowed his name to be placed as consulting oculist, and gave the public confidence, in this infant establishment, by officially announcing, in the fourth annual Report of the London Eye Infirmary, the instructions which he had afforded me,* although this institution was in a situation commanding the practice of the whole of the west of England; and he knew, that, in obedience to the injunctions of some of its first and best patrons, I had been reluctantly compelled to relinquish the general practice of surgery and medicine, for the same particular branches which he had himself chosen. This was the man who has been accused of illiberal concealment!

Having been his constant, indeed his sole, assistant, (the two gentlemen who, after I left his tuition, attended the practice of the infirmary for three months each, not having been admitted to witness his operations for cataract,) I can bear testimony to the successive alterations in his practice; while, as, justly observed by the respectable editor of his posthumous work, in a letter inserted in the last Number of this Journal, "with a head to devise, and a hand to execute, he was deliberately following up all the possible means of

---

* "This process for curing the cataract in children, together with other observations relative to the eye, which I am about to publish as soon as the necessary arrangements can be made, has already been freely communicated to an individual, and the ample scene of experience which this Infirmary affords opened to his view, from a disinterested wish to promote his professional object. Mr. Adams has since settled in Exeter, and there established a charity on the model of this institution. This event I could not refrain from noticing, because it must excite in your minds, and the minds of the governors, the grateful reflection that your benevolence has given life and activity to an institution which has benefited society not only in its own operation, but by giving direct origin to an establishment producing its contingent of good in another part of the kingdom. That which was so liberally given in the spirit of private friendship, has been so long withheld from the public, in the hope of making it more worthy of acceptance, and not through a mercenary motive, as some have malignantly observed, or an inclination to boast the possession of a secret." Vide Mr. Saunders's official Letter, inserted in the 4th Annual Report of the London Eye Infirmary, published in March 1809.

operating operating for the cataract, to decide, by a just comparison, their relative advantages." This was the original cause of the delay so much complained of in the publication of his practice; and the fatal disorder that so long preyed on his constitution, at length terminated his existence before he could fulfil the promise he had given. No person felt more regret at the delay in the appearance of Mr. Saunders's posthumous work than myself, as I was thereby deterred from publishing some important improvements which I had made in his instrument, and the operation for cataract, under the impression, that they would, in some degree, have anticipated his work, which I should have considered a breach of good faith and gratitude which could not be sanctioned by any personal advantages, particularly as the proceeds of its sale had been generously and exclusively appropriated to the benefit of his widow, by the committee of the London Infirmary for curing Diseases of the Eye.

From the period I found it necessary to abandon the posterior operation taught me by Mr. Saunders, it has been my invariable custom, wherever called upon to operate on diseases of the eye, to invite my professional acquaintance to witness my modes of practice, that they might be enabled to judge of their comparative merit with those in general use. Had I, however, possessed the prudent caution of my deceased friend, I should not have been anticipated in communicating to the public the revival of Cheselden's obsolete operation for artificial pupil, or have seen an imperfect description given of one of my operations for the cure of cataract, circumstances which *obliged me* to publish my practical observations much sooner than I should otherwise have done after the appearance of Mr. Saunders's book; neither should I now feel it necessary, but from some other effects of that incaution, to repeat the description already given in my work, of my instrument and operation for the cure of the solid cataract in children and adults, which for nearly *four years past* I have made use of and practised; or have thought it requisite now to give the history of their origin.

In the second section of my Practical Observations on Cataract, is given the following description of my two-edged needle, and mode of conducting an operation for the cure of the solid cataract in children and adults:

"The blade of the needle which I now employ is eight tenths of an inch long, the third part of a line in width, nearly flat, having a slight degree of convexity throughout its whole extent. It is spear-pointed, with the edges made as sharp as possible to the extent of four-tenths of an inch. Beyond the cutting edges it gradually thickens, so as to prevent any discharge of the aqueous or vitreous humor. The handle is of the usual length. The effect of this instrument is easily understood: it enters the coats of the eye with the most perfect freedom, traverses the posterior chamber without in the least degree displacing the cataract, and cuts it in pieces with great ease when it is not very hard. Instead of the cutting-edge being confined to the mere point, it extends so far back as to be nearly, if not entirely, equal to the diameter of the cataract; by which, in children and adults, I am frequently enabled, at the first operation, to cut in pieces both the capsule and opake lens. Its convexity also affords a sufficient degree of strength to prevent it from bending during its passage through the coats of the eye, should they present any considerable resistance; but it cuts so very sharp, that this, in general, is not to be feared. Some caution in its use, however, is requisite on the part of the operator; otherwise, as it is an instrument of great power, and traverses the eye with so much facility, it may wound the iris, or its point may be carried too far towards the nose." Pages 128 and 129.

"The pupil being in a dilated state, from the application of the extract, or a strong solution of the belladonna within the eye-lids, about an hour previous to the operation, and having secured the eye by a gentle pressure with the concave speculum,* introduced under the upper eyelid, I enter the two-edged needle through the sclerotic coat about a line behind the iris, with the flat surface parallel to that membrane. I then carry it cautiously through the posterior chamber, without, in the slightest degree, interfering with the cataract or its capsule, till the point reaches the temporal margin of the pupil, when I direct it into the anterior chamber, and carry it on to the nasal margin of the pupil, in the line of the transverse diameter of the crystalline lens. I then turn the edge backwards, and with one stroke of the instrument cut both capsule and cataract in halves. By repeated cuts in different directions, I afterwards divide the opaque lens and its capsule in many pieces, and at the same time take particular care to detach as much of the latter as possible from its ciliary connection. As soon as this is accomplished, I turn the instrument in the same direction as when it entered the eye, and with its flat surface bring forward as many of the fragments as in my power, into the anterior chamber, by which means I frequently leave the upper part

* For a description of this new instrument, see, in my work, page 131, and the representations of its form, Plate 3, Fig. 6 and 7.

of the pupil perfectly free of opacity. By cutting in pieces the capsule and lens at the same time, not only is capsular cataract generally prevented, but the capsule is aslo much more easily divided into minute portions, than when its contents have been previously removed. The central division of the capsule and cataract, prevents the liability of their becoming too soon detached from the ciliary processes, and revolving on the needle, or slipping undivided into the anterior chamber. In cases of large and solid cataract, this is very essential to the perfect execution of the operation, as the natural connection of the capsule with the ciliary processes, assists, in conjunction with the vitreous humor, to afford a sufficient counter-resistance, to admit of its nucleus being completely divided; which, were that connection detached, could not be divided but by repeated operations." Page 134, &c.

It will be considered rather a singular circumstance, that the same case which first suggested to me the revival of Cheselden's obsolete operation for artificial pupil, should also have given origin to the instrument and modus operandi I have just described.

"William Pike, of Widworthy, near Honiton, Devon, was admitted a patient of the West-of-England Infirmary for curing Diseases of the Eye, in November 1808. He was effected with capsular cataracts, which had succeeded an unsuccessful operation of extraction, in a general hospital. The pupil of each eye was nearly obliterated, and the rays of light were prevented from passing through the small aperture which still remained, by portions of capsule, thickened, and firmly adhering to the posterior part of the iris. I at first attempted, with the flat part of my needle, to separate the adhesions, but, finding this impracticable, I *turned the instrument edgeways, and with the point cut through the capsules*, thereby making an aperture in each, sufficiently large to enable the man to see the minutest objects with the assistance of proper glasses."

At this period (Nov. 1808) I used Mr. Saunders's needle, which was spear-pointed, but which did not cut further back than the shoulders of the spear, as he was then content to bring merely the point into action. The fortunate result of the operation on Pike, encouraged me to hope, that, with one of my cataract needles, made very sharp, I might succeed in a similar manner in dividing the fibres of the iris in a case of closed pupil.

I immediately gave directions to a surgeon's instrument maker at Exeter (Mr. Shorto) to alter one of Mr. Saunders's needles—to thin it back considerably further towards the handle, and to make it to cut for the space of three or four lines lines from its point "as sharply as a lancet." With an instrument thus altered, in Dec. 1808, and assisted by my nephew and apprentice Mr. Hockin, I operated upon a patient in the infirmary of the name of Heard,* whose pupil had become entirely obliterated in consequence of extraction having been unsuccessfully performed. The operation I performed on him was very much the same as that recommended by Cheselden; and, as soon as the iris was divided, its radiated fibres contracted, and formed an opening of a large size, nearly circular, and quite clear.

As the facility which I experienced in Pike's case in dividing the thickened capsules with the sharp point of Mr. S.'s needle, when turned horizontally, suggested to me the advantage of altering the instrument, so the ease with which I divided the iris in Heard's case with the instrument thus altered, induced me to try if I could not with it also cut in pieces a solid cataract in the manner already described; and my success was such as to justify me in the adoption of the instrument and operation ever since, in cases of solid cataract in children and adults. I have even frequently succeeded in persons of the most advanced age, an instance of which occurred in the case of John Taylor, seventy-two years of age, who was admitted as an in-patient of the West-of-England Eye Infirmary, in March 1809, when, assisted by Mr. Hockin, I cut the whole of the lens and capsule (of one eye) into pieces, and lodged them thus divided in the anterior chamber. See case 36 in my work.

Soon after the adoption of this instrument and operation, I gave Mr. Saunders a description of them, communicated the power which I found the needle to possess when used in the manner described, and strongly recommended its adoption in cases of *elastic* cataract, in which description of disease I had known him sometimes to have been obliged to repeat his operation six or eight times. He then objected to it, apprehensive that, possessing so much tenuity, it might break in the eye.† By the eighth plate in his posthumous work,

---

* From a typographical error in my work, which, until it had gone through an impression, escaped unnoticed, Heard is there stated to have been operated on for artificial pupil in Dec. 1809, instead of the same month the preceding year, as will be seen by the second case of artificial pupil, Richard Bligh, on whom I operated April 1809.

† At this period I had not succeeded in getting a needle of the exact form I wished, as the surgeon's instrument maker at Exeter, and Mr. Smith, of St. Saviour's Church Yard, Southwark, (the latter of whom has worked for the West-of-England Eye Infirmary from its first work, he, however, afterwards appears to have partially adopted the alteration I had repeatedly urged, as it represents his needle to cut back from the shoulders of the spear to an extent of two lines or upwards.

During the years of 1809 and 1810, Samuel Frederick Milford, esq. Vice-president of the West-of-England Infirmary for curing Diseases of the Eye, and many of the committee and governors of that institution, were in the frequent habit of witnessing my different operations for the cure of cataract, and examining the instruments with which they were performed. Being professionally called to Bath in the spring of the latter year, among many other patients laboring under cataract, I operated on Mrs. C. (case 39) in the presence of Dr. Wilkinson, of that city, and several other professional gentlemen. In this case "I divided (at once) the whole of the cataract and its capsule very freely, but suffered them to remain *in situ*," which I accomplished with my two-edged needle in the usual manner. I was at that time requested, by the committee of the Blind Asylum at Bristol, to operate on some persons belonging to that institution, one of whom, a girl about twelve years old, labored under a solid cataract. In the presence of Dr. Pope, several surgeons of that city, and a considerable number of the members of the committee, I performed the operation with my two-edged needle precisely in the manner described, after which Dr. Pope took a drawing of the eye, for the purpose of forwarding it to a gentleman in Manchester, an intimate friend of that late eminent surgeon Mr. Gibson.

At the official request of the committee of the Infirmary for curing Diseases of the Eye, at Exeter, his Royal Highness the Prince Regent was graciously pleased to nominate me his Oculist Extraordinary, and I was, in consequence, summoned to London from Bath, in May 1810, to receive the appointment. During my stay in town, assisted by Mr. John Ring, late of South Molton-street, I operated, in my usual manner, and with the two-edged needle, on Mr. J. F. nephew to the late Right Hon. Lord F. who labored under solid congenital cataracts.

A few days after this (in the beginning of June), I invited Mr. Ware, with some other professional gentlemen, to witfirst establishment) informed me they were unable to make it cut as sharply as I directed, without reducing the blade to this degree of tenuity. My present needle is, however, entirely exempt from this objection, a pattern of which I have left with Mr. Smith, and also of my concave speculum, as well as the other instruments recommended in my work.

ness ness the operation on Mr. Purkis, of Chancery-lane, a gentleman thirty years of age, who labored under the elastic congenital cataracts. On this occasion I performed the operation with a one edged knife, being disappointed by the instrument maker in obtaining the needle which I prefer in such cases. Mr. Ware with great candor acknowledged to the several gentlemen present, and after I left London to the patient and his friends, at his several visits for the purpose of observing the progress of the cure, and ascertaining the after-treatment which I had directed to be pursued, that he had never practised, or before seen, a similar operation, of which he *then* expressed his unqualified approbation. In a third edition of the second volume of his work on Diseases of the Eye, published in Nov. 1811, he has given an imperfect description of this operation, and mentions that he and his son have successfully practised it with a similarly constructed instrument to that which he had seen me use. He strongly recommends my operation for the cure of congenital cataract in young children, thereby admitting its manifest advantage over extraction, which, in the *former* editions of his works, he advises not to be performed in such cases until the patient had attained an age when he could understand the necessity of keeping his eyes steady. His work is, however, calculated to excite an erroneous opinion of my practice for the cure of the solid cataract in "adults and old persons;" because, notwithstanding that he was then entirely unacquainted with my operation and instrument for "old persons," both of which are very different from those he saw—that he had never seen my two-edged needle "for adults"—and that he was apparently ignorant of the precise manner in which I performed the operation,* he still declares his decided preference, from the experience of thirty years, to the operation of extraction in such cases.† I am, however,

---

* "The intention that Mr. Adams had in view evidently was like that described by Mr. Hay and Professor Scarpa, to lacerate the capsule, and bring as many of the pieces as could be collected together into the anterior chamber." Vol. ii. page 368.

† In the edition of this volume in 1811, and also in a small pamphlet extracted from it, Mr. Ware has entirely changed the opinion given in the two former editions of his works (the last published in 1805) as to the efficiency of Cheselden's operation for artificial pupil, an operation which he for the first time recommends; but it escaped his recollection to mention, that I had, *more than eighteen months* before, communicated to him, in consultation on the case of Lady W. (who has allowed me to make use of her name in this question, should however, highly gratified to find that Mr. Ware since appears, by his practice, to approve of the use of the needle in "adults and old persons;" if I may be allowed to judge from two of his patients whom I have lately seen. One a gentleman thirty years old, in one of whose eyes he has successfully operated for a solid cataract in the same manner in which he has described my operation; the other an old man of fifty, on whom Mr. W.'s son operated with the needle in his father's presence, and cut in pieces the solid cataracts under which the man labored.

At an interview I had with Mr. Ware at his house, previous to the operation on Purkis, he shewed me some opake and thickened capsules preserved in spirit, which he had extracted, and then expressed his firm belief, that, although the aqueous humor was sufficiently solvent to remove an opake lens (which he had proved in some cases with the needle), yet that it was totally inadequate to the solution of the capsule when thus diseased.* I am inclined to think it was under this impression that he was in general deterred from operating with the needle on children laboring under congenital cataracts, or that, when he did venture to deviate from his usual practice, he so frequently failed in removing the crystalline capsule when thus morbidly affected, as proved by cases 19, 20, 21, 22, 25, 26, and some others which I have detailed in my practical Observations on Diseases of the Eye.†

But it be required) the successful result of two cases of this kind in the West-of-England Eye Infirmary, when he almost discredited my assertions, so inefficacious did he *then* consider the operation of Cheselden. See the first chapter on Artificial Pupil in my Practical Observations.

* "If, in addition to the opacity of the crystalline humor, its capsule be also opake, either in its anterior or posterior portion, or in both, (which circumstance cannot be ascertained before the operation,) and, in consequence of this, the operation above-mentioned should not prove successful, it will not preclude the performance of extraction afterwards if this be thought adviseable." Mr. Ware's works on Diseases of the Eye, vol. ii. page 380. See also the history of the Extraction of an Opake Capsule after its contents had been removed by the previous use of the Needle, page 382 of the same volume.

† I have recently operated for capsular cataract on the son of the Rev. Mr. W. of Castle Cary, Somerset, whose case Mr. Ware has published both in the second volume of his works, and in the Philosophical Transactions, as a parallel to Cheselden's celebrated case of congenital cataracts cured at the age of fourteen. I found the

But, to return to the history of my operation for cataract in children and adults—Early in October, 1810, I addressed an official letter to the committee of the West-of-England Eye Infirmary, informing them of my intended resignation, in which, alluding to some improvements I had made in my practice on diseases of the eye, since the establishment of that infirmary, I expressed myself in the following manner: "In consequence of having also made an alteration in the instrument for curing cataract, as well as in the method of conducting the operation taught me by my late most highly-respected friend Mr. Saunders, I am enabled to succeed in a much shorter space of time than I was in the earlier periods of my practice; the necessity of repeating so frequently the operation to effect a permanent cure, which then existed, being by these means almost entirely superseded. The result of my experience in cases of this nature, leads me to assert, that no operation of importance in surgery is more uniformly successful than that which several of my professional friends have seen me practice in the cure of cataract for *these last eighteen months*, having, within the last twelve months, operated on more than forty cases successively, in my public and private practice, without experiencing one unfavorable result."—"In my official letter to the committee published in the last year's Report, I intentionally avoided making any allusion to these improvements, from my anxiety to avow to the world, in the *fullest manner possible*, the obligation and gratitude I felt towards my ever-lamented preceptor and friend Mr. Saunders."

This letter was inserted by order of the committee, in the second Annual Report of the Institution, published in October 1810; and, on quitting Exeter in the following month,

---

right eye of Mr. W. completely destroyed by an unsuccessful attempt made by Mr. Ware to extract the opake capsule sometime after he had removed its contents by the use of the needle. After the pupil of the left eye had been expanded to the utmost by the application of the belladonna, for the purpose of having a drawing taken of the appearance of the opake and thickened capsule, by his cousin, Mr. Woodforde (the eminent artist), I repeatedly examined the eye most minutely, as did Mr. W. and my nephew Mr. Hockin; but we could not perceive any part of the pupil clear. The small portion of light admitted to the retina appeared to pass through the upper edge of the capsule, which was much less opake than any other part. By putting large letters within an inch of his eye in an oblique direction, he could name one at a time, and could tell the hour, &c. by his watch, but he was unable to see sufficiently to walk without a guide, although the operation had been performed ten or twelve years.

I left

I left behind me several sets of my two-edged cutting needles belonging to the Infirmary.

In March 1811, I was requested to go to London from Bath, where I then resided, to operate on the daughter of a gentleman in Portman-square, laboring under congenital cataract. I operated on one eye in presence of the eminent accoucheur Mr. Croft, and of Mr. Chilvers, surgeon, in Upper Burlington-street, to whom I pointed out in what consisted the peculiarities of my operation, and shewed them my needle, which they examined very minutely. A few days after, my nephew, Mr. Hockin, left one of my two-edged needles, as a pattern, at Messrs. Saviguy's, by which he was ordered to alter some others.

It appears that Mr. Savigny's partners have been in the habit of exposing for sale different eye-instruments which I have invented, and some even before the design I had in view was perfected. These liberties were taken in direct opposition to my wishes and repeated directions, as I was unwilling to be anticipated in my claims to their invention.* Yet so little were these repeated directions attended to, that, when I came to reside in London, in November 1811, I found my present improved artificial pupil knife also exposed for sale, although I had apprised him, in my former visit to town, of my intention of publishing on the subject of artificial pupil, when I should particularly describe that instrument.

Being called to Dublin, in the summer of 1811, by a gentleman, for the purpose of operating on three of his children who had congenital cataract, my operations and instruments while there, were seen and examined by most of the respectable professional gentlemen in that city. In autumn, on my return to England through Edinburgh, I had there the honor of being officially requested by the surgeons of the Royal Infirmary, to demonstrate some of my operations for cataract on patients in that institution, which I did in the presence of several of the professors, and most of the students of the university; and in compliance with the wishes of Mr. Russell, the professor of Clinical surgery, I gave a *written description* of the operation in question, which he read to the numerous class of students in the Infirmary;

* During this stay in London, having requested Mr. Savigny's partner not to continue to exhibit one of my artificial pupil knives, which he had previously made according to my directions, he told me he had been informed by a gentleman *(whose name I shall not now mention)*, that its peculiarities did not originate with me, but "that it was Mr. Saunders's instrument."

when he also exhibited my double-edged needle, a pattern of which, and of my other instruments for the cure of cataract, and the formation of an artificial pupil, I left with the surgeons' instrument maker of the Infirmary, at the request of its senior surgeon, my highly-esteemed friend, Mr. George Bell.

I trust I shall be pardoned for having given this circumstantial detail of my professional proceedings, which, I confess, under other circumstances, might be justly deemed ostentatious; but I have thought it necessary, as, by doing so, I have, by the priority of dates, and respectability of references, *fully established my indisputable claim to the invention of the instrument and operation*, which are the subject of this paper. I have the honor to remain, Gentlemen,

Your obedient humble Servant,

WILLIAM ADAMS,

Member of the Royal College of Surgeons in London, and the late Surgeon to the West-of-England Infirmary for curing Diseases of the Eye, instituted at Exeter.

28, *Albemarle-street, December* 1812.

