# Corrigenda

**Published:** 1813-03

**Authors:** 


					Pa"e 101, line 7, for their orifices, rend the posterior orifice.
41, for in, read on.
Page 104, line 2, for deduction, read reduction; and bsfore frov>}
insert a mark of parenthesis.

				

