# Corrigenda

**Published:** 1813-03

**Authors:** 


### *CORRIGENDA.*

Page 89, for *tenacious*, read *cautious*.

